# Space-time clustering of nasopharyngeal carcinoma in Greenland Eskimos.

**DOI:** 10.1038/bjc.1985.276

**Published:** 1985-12

**Authors:** H. Albeck, M. Coleman, N. H. Nielsen, H. S. Hansen, J. P. Hansen

## Abstract

Evidence of epidemicity of nasopharyngeal carcinoma (NPC) was sought in Greenland Inuits, who have a high incidence of this cancer, by examining the births of NPC cases for evidence of clustering in time and space. Births of cases were concentrated in autumn and winter. Fifty-four cases were analysed, and a two-fold excess of clustering within one year was observed, both within single districts and between adjacent districts. This excess was not significant at the 5% level; about 90 cases would have been required to confirm the observed effect at this level of significance. It is suggested that a search for space-time clustering of NPC cases in larger high-risk populations might prove more fruitful.


*Br. J. Cancer* (1985), **52**, 909–914

### Space-time clustering of nasopharyngeal carcinoma in Greenland Eskimos

H. Albeck[1], M. Coleman[2], N.H. Nielsen[3], H.S. Hansen[4] & J.P.H. Hansen[5]

[1]*Ear, Nose and Throat Department, Righospitalet, Blegdamsvej 9, DK-2100 Copenhagen, Denmark;* [2]*Imperial Cancer Research Fund, Gibson Builiding, Radcliffe Infirmary, Oxford OX2 6HE, UK;* [3]*University Institute of Forensic Medicine, Frederik 5's vej, DK-2100, Copenhagen, Denmark;* [4]*Department of Head and Neck Oncology, Radiumstationen, Finseninstitutet, Strandboulevarden 49, DK-2100, Copenhagen, Denmark;* [5]*Department of Pathology, Gentofte Hospital, DK-2900, Copenhagen, Hellerup, Denmark.*

**Summary** Evidence of epidemicity of nasopharyngeal carcinoma (NPC) was sought in Greenland Inuits, who have a high incidence of this cancer, by examining the births of NPC cases for evidence of clustering in time and space. Births of cases were concentrated in autumn and winter.

Fifty-four cases were analysed, and a two-fold excess of clustering within one year was observed, both within single districts and between adjacent districts. This excess was not significant at the 5% level; about 90 cases would have been required to confirm the observed effect at this level of significance. It is suggested that a search for space-time clustering of NPC cases in larger high-risk populations might prove more fruitful.

Nasopharyngeal cancer is a rare tumour in most populations, with an annual incidence below 1 per 100,000, but in various ethnic Chinese populations the annual incidence rate is ten to twenty times higher, and reaches 29 per 100,000 in Cantonese living in Singapore (Shanmugaratnam, 1982). Most nasopharyngeal carcinoma (NPC) in these high-risk areas is undifferentiated squamous carcinoma (WHO type II and III) (Clifford & Beecher, 1964; Schmauz & Templeton, 1972; Nielsen *et al.*, 1977; Cammoun *et al.*, 1978).

The ethnic and geographical distribution of NPC appears to indicate that both genetic and environmental factors are important in its aetiology. There is an increased risk of NPC in migrants, whether Caucasian or Chinese, who are born in high risk areas, even though they live most of their later life in low risk areas. This suggests that any environmental factors in the subsequent development of NPC act during infancy or early childhood (Zippin *et al.*, 1962; Buel, 1973, 1974).

There is now both serological and cellular evidence (Anderson-Anvret *et al.*, 1978; Klein, 1979; Saemundsen *et al.*, 1982) in support of the association reported by Old *et al.* (1966) between undifferentiated NPC and Epstein–Barr virus (EBV). Cases of NPC show high serological reactivities against all four major EBV antigens, with higher titres in more advanced cases, whilst there is no such pattern in other tumours of the nasopharynx, or in tumours of adjacent tissues (de Thé *et al.*, 1978). A high content of DNA from the EBV genome has been found in malignant epithelial cells from nasopharyngeal carcinoma (Klein, 1979).

NPC is relatively common in Greenland Eskimos (Nielsen *et al.*, 1977), with annual incidence rates in 1968–1972, adjusted to world standard population (Doll *et al.*, 1970), of 12.3 and 8.5 per 100,000 in males and females, respectively. These rates are about twenty times higher than the rates in Denmark (Nielsen & Hart-Hansen, 1982). The EBV genome has been demonstrated in malignant NPC epithelial cells from Greenland Eskimos (Saemundsen *et al.*, 1982). Seroconversion to EBV in Greenland is almost universal by the age of two years (Albeck *et al.*, 1985), indicating early exposure to EBV infection. On the basis of the seroepidemiology of EBV (Henle & Henle, 1979), it can be assumed that primary infection with EBV also took place during infancy and childhood during the first half of this century, when the subjects in this study were born.

If EBV infection is causally related to nasopharyngeal carcinoma, however, then NPC might show cyclical epidemicity in response to waves of EBV infection, or to variations in the oncogenicity of the virus. Space-time clustering of NPC cases would be evidence of such epidemicity. Familial clusters of NPC have been observed (Shanmugaratnam, 1982), but there has been no report of NPC clustering in a population.

We have therefore sought evidence of space-time clustering of NPC in the Inuit population of Greenland, with the prior hypothesis that cases may share a common oncogenic exposure to EBV in infancy. For a disease with a very long latency between exposure to the causal agent and clinical diagnosis, individual latent periods would be

Correspondence: H. Albeck.
Received 16 April 1985; and in revised form, 30 July 1985.

© The Macmillan Press Ltd., 1985

expected to vary considerably, and clustering at the time of *diagnosis* might be very weak, even if the causal exposures were highly clustered. We therefore looked for evidence that NPC cases were *born* closer together in time and space than would be expected by chance, regardless of when the NPC was actually diagnosed.

### Study population

The native Greenland population is of Inuit Eskimo origin, with considerable Caucasian admixture (Kissmeyer-Nielsen *et al.,* 1971). The Inuit population increased from $\sim$10,000 at the turn of the century to 16,000 in 1930, and 42,000 in 1982. Between 1900 and 1982, the annual number of births rose from about 500 to 1,000 (Bertelsen, 1937; Ministry for Greenland, 1983).

During the period from 1900–1955, during which the births in this study took place, the typical Inuit family comprised 6–10 persons living in their own detached home, built of stone and turf, and averaging about $9 \times 12$ feet ($10\,m^2$) in ground area and about 6 feet (2 m) in height. All family members, and guests, would sleep in the same bed (about $6 \times 5$ feet). The general standard of hygiene was poor (Bertelsen, 1937), and infectious diseases spread easily.

The country is divided into 16 medical districts, each with its own small hospital based in the coastal town, which is the local trade and social centre. All medical care is free; cancer patients are usually referred to Denmark for investigation and treatment. All hospital records in Greenland were checked for the period 1950–1983. Records for all patients transferred to Denmark were also reviewed. The files of the Danish Cancer Registry in Copenhagen were searched for NPC cases in Greenland residents. Registrations of lymphoma in cervical lymph nodes or of metastases in those nodes from an unknown primary were also reviewed, to exclude NPC. Biopsy specimens and reports from all these patients were reviewed. Data for all patients with NPC were extracted from hospital records.

### Method

Knox's method (1964) was used to determine if cases of NPC showed evidence of space-time clustering at birth, compatible with a shared oncogenic exposure in infancy. The spatial $(X, Y)$ coordinates assigned to each case were those of the central settlement in the district of birth, and defined as the distance in miles east and north, respectively, of an imaginary origin to the south-west of Greenland (see map, Figure 1). Cases born in the same settlement were thus given the same spatial coordinates. Three space-intervals were then chosen for assessment of clustering: 1 mile, 100 miles and 1,000 miles. Two cases born 'within a mile' of each other would then be born within the same district. Given the distribution of settlements in Greenland, and the predominantly coastal movement between settlements imposed by the terrain, these distances were intended to reflect spatial clustering within single districts, between neighbouring districts and within Greenland as a whole, respectively.

The time coordinate assigned to each case was the subjects date of birth. Five time intervals were chosen arbitrarily, since there was no clear basis for supposing that cases born within any particular time interval should be considered close in time. These intervals were 3 and 6 months, and 1, 2 and 3 years. Both time and space intervals were chosen before carrying out any analysis of the data.

All possible pairs of cases were examined (for $N$ cases there are $N(N-1)/2$ pairs) to see whether they were born close to each other in time and space. The observed number of such close pairs is assumed to be drawn from a Poisson distribution with a mean equal to the expected number of pairs. The expected number of pairs is calculated under the null hypothesis that the temporal distribution of births is independent of their distribution in space. Any excess of observed close pairs over the number expected is then tested by reference to tables of the Poisson distribution. This test is conservative if the number of close pairs is large. Fifteen tests were performed, one for each combination of the 3 space intervals and 5 time intervals; these tests are not independent, since close pairs observed within any given limits will automatically include all close pairs observed within more stringent limits.

The analysis was carried out with a computer program by Pike and Bull (1974).

### Results

Fifty-six incident cases of NPC in Greenland Inuits diagnosed between 1 January 1950 and 30 June 1983 were collected. Two cases, born before 1900, were excluded because of uncertainty about date of birth. Of the remainder, 51 had firm histological evidence of NPC (50 undifferentiated squamous, 1 differentiated squamous) and in 3 cases the diagnosis was clear-cut on clinical grounds alone. We believe we have included all incident cases of NPC in Greenland Inuits recorded during the 33 years covered by the study.

**Figure 1** Map of Greenland, showing the main settlements and the number of NPC cases born there. The figures beside each settlement show the intervals, to the nearest month, between successive births (in the period 1900–1955) of persons later diagnosed with NPC (1950–1983). Each case is represented by a dash (—).

The median age at diagnosis of NPC was 51.5 years. Most cases (80%) were born in the period 1900–1929. The distributions of NPC cases by month and period of birth and by age and period of diagnosis are given in Table I. There is a marked seasonal pattern to the births of NPC cases, with a peak in autumn and a trough in spring (Figure 2). This was an unexpected finding, and to pursue it we used Edwards' (1961) harmonic analysis, which tests for departure from the null hypothesis that there is no seasonal peak, and fits a sine wave to the observed distribution. There was an almost six-fold difference between the fitted peak and trough frequencies, in October and April (7.7 and 1.3; peak-to-trough ratio 5.9). Addition of one

**Figure 2** Seasonal distribution of births of NPC cases ($n = 54$). The curve is a sine wave fitted by Edwards' (1961) method (see text).

**Table I** Distribution of 54 NPC cases by month and period of birth, and by age and period of diagnosis.

| *Month of birth* | | *Period of birth* | | *Age at diagnosis* | | *Period of diagnosis* | |
|---|---|---|---|---|---|---|---|
| January | 4 | 1900–4 | 4 | 20– | 2 | 1950– | 2 |
| February | 2 | 1905–9 | 6 | 30– | 6 | 1955– | 2 |
| March | 4 | 1910–4 | 7 | 40– | 13 | 1960– | 4 |
| April | 0 | 1915–9 | 6 | 50– | 22 | 1965– | 7 |
| May | 3 | 1920–4 | 8 | 60– | 10 | 1970– | 13 |
| June | 4 | 1925–9 | 12 | 70+ | 1 | 1975– | 13 |
| July | 7 | 1930–4 | 5 | | | 1980–3 | 13 |
| August | 3 | 1935–9 | 1 | | | | |
| September | 6 | 1940–4 | 2 | Median age at diagnosis 51.5 | | | |
| October | 7 | 1945–9 | 1 | | | | |
| November | 6 | 1950–5 | 2 | | | | |
| December | 8 | | | | | | |

**Table II** Space-time distribution of births (1900–1955) of 54 NPC cases diagnosed 1950–1983.

| | *Observed* (*O*) and expected (*E*) *numbers of case pairs born within: TIME* | | | | | | | | | | | | | | | |
|---|---|---|---|---|---|---|---|---|---|---|---|---|---|---|---|---|
| | *3 months* | | | *6 months* | | | *1 year* | | | *2 years* | | | *3 years* | | | |
| *Space* | *O* | *E* | *P*[b] | *O* | *E* | *P* | *O* | *E* | *P* | *O* | *E* | *P* | *O* | *E* | *P* | *All times* |
| Same district[a] | 3 | 1.5 | 0.18 | 6 | 3.2 | 0.10 | 9 | 5.6 | 0.11 | 14 | 11.8 | 0.29 | 17 | 16.3 | 0.47 | 123 |
| 100 miles | 4 | 2.3 | 0.19 | 8 | 4.9 | 0.12 | 11 | 8.6 | 0.25 | 20 | 18.1 | 0.36 | 24 | 25.1 | 0.61 | 189 |
| 1000 miles | 17 | 16.5 | | 36 | 35.9 | | 62 | 63.0 | | 133 | 132.8 | | 185 | 184.2 | | 1387 |
| All distances | 17 | | | 37 | | | 65 | | | 137 | | | 190 | | | 1431 |

[a]Cases born within the same district were given the same spatial co-ordinates: see text; [b]One-sided Poisson *P*-value.
*Note:* For 54 cases, each of 1431 possible case pairs (54 × 53/2) is examined. The 3 close pairs born within the most stringent limits (within 3 months of each other, within the same district) are included among observed close pairs defined by an any less stringent limits. Each number in the body of the Table therefore includes all those given to the left or above.
In the calculation, time intervals were defined as less than or equal to 91, 182, 365, 730 and 1095 days, respectively.

imaginary birth in April, in order to assess distortion due to the absence of observed births in this month, caused some flattening of the fitted sine wave (peak 7.0, trough 2.2, ratio 3.2), but the mid-October peak was unchanged. This analysis was prompted by the observed data rather than any prior idea about a seasonal pattern of births, and *P*-values testing departure from the null hypothesis are therefore not reported.

The spatial distribution of the births of NPC cases is shown on the map (Figure 1), which also shows, for each district, the number of cases born there and the interval between the births of successive cases, to the nearest month.

The observed and expected numbers of close pairs revealed by the clustering analysis are shown in Table II for each combination of space and time limits. Among 54 cases, there are 1,431 possible pairs (54 × 53/2). There were, for example, 17 pairs born within 3 months of each other, and 123 paris born within the same district; 3 pairs met both criteria, compared to 1.46 pairs that would have been expected if there were no association between the times and places of birth of the cases (17 × 123/1,431).

The observed number of pairs born close in space and time was about twice the expected number over short time intervals (less than one year), both within the same district and between adjacent districts, but none of the excesses is significant at the 5% level. At time intervals greater than one year, there is no evidence of clustering. There is no evidence of clustering within Greenland as a whole (1,000 miles) at any time interval.

None of the close pairs involved either the case with differentiated squamous carcinoma, or any of the three cases diagnosed on clinical grounds alone.

### Discussion

Considerable efforts were made to find all recorded cases of NPC in Greenland, but the small number of cases before 1950 suggests that some may have been missed, perhaps misdiagnosed as tuberculosis, which was the major health problem in Greenland until that time, and which, in its terminal stages, may share with NPC the clinical features of cervical lymphadenopathy, cranial neuropathies and cachexia. Medical facilities have also improved greatly since 1950, and ascertainment of cases is probably higher. Infant mortality has fallen steeply since the early part of the century, however, even though life expectancy at birth in 1950 was still only 28.5 years, so part of the recent increase in cases of NPC may be real, reflecting the demographic shift to an older population since 1950. During the period covered by the births of cases in this study, 1900–1955, there were no dramatic changes in the relative birth rate of different districts, which might have produced a spurious clustering of births.

The small excess of clustering of NPC cases at birth observed in this study is suggestive of a short term, local effect, but while these results are consistent with epidemicity of NPC due to a shared oncogenic exposure at or near birth, they do not provide strong evidence. For the observed two-fold excess of clustering within 3 months and the same district to reach the 5% level of significance, given the observed distribution of cases in time and space, about 90 cases would have been required.

Some of the choices made in the analysis may merit comment. The time periods chosen were long enough for considerable movement within the district to have occurred, with maximal opportunities for contact in the central settlement, which is the social centre of the district. The pattern of poliomyelitis, scarlet fever and meningitis epidemics during 1900–1930 shows that entire districts were affected within a few months, whereupon neighbouring districts became affected (Bertelsen, 1943). The district was therefore chosen as the smallest spatial unit of clustering, and the central settlement as the locus of each case born in that district.

The seasonal variation in births of NPC cases is surprising. No seasonal variation in EBV infection has been observed in Greenland, where almost all children now acquire serological evidence of EBV infection between 5 months and 2 years of age. Since EBV infection is almost universal, and only a tiny proportion of those infected will develop NPC, it is clear that the virus is not a sufficient cause of the carinoma. Given a latency of 40 or more years between EBV infection and development of NPC, establishing any connection retrospectively may prove difficult. Other theories of NPC aetiology include consumption of salted fish containing nitrosamines in early life (Ho, 1972). Traditionally, Eskimo food has included dried meat and anaerobically fermented meat and fish. Hirayam and Ito (1981) have recently advanced the theory that tumour promoters in traditional Chinese herbal medicines may interact with EBV in the aetiology of NPC.

Other populations at high risk of NPC are much larger than the population of Greenland Inuits, and would permit collection of a much larger number of cases in a shorter time; ascertainment of more recent cases should be more complete, and data on other exposures could also be obtained. The small but intriguing excess of space-time clustering observed here suggests that a search for this effect in a larger high-risk population might prove more fruitful.

### References

ALBECK, H., BILLE, T., FENGER, H.J. & others. (1985). Epstein–Barr virus and serological profile in Greenland Eskimo children. *Acta Paediat. Scand.* (in press).

ANDERSON-ANVRET, M., FORSBY, N. & KLEIN, G. (1978). Nasopharyngeal carcinoma. *Prog. Exp. Tumor Res.*, **21**, 100.

BERTELSEN, A. (1937). Grønlandsk medicinsk Statistik og Nosografi. II Sundhedsvilkaarene i Grønland. Reitzel, Copenhagen.

BERTELSEN, A. (1943). Grønlandsk medicinsk Statistik og Nosografi. IV Akutte infektionssyngdomme i Grønland. Reitzel, Copenhagen.

BUEL, P. (1973). Race and Place in the Etiology of Nasopharyngeal Cancer. *Int. J. Cancer*, **11**, 268.

BUEL, P. (1974). The effect of migration on the risk of nasopharyngeal cancer among Chinese. *Cancer Res.*, **34**, 1189.

CAMMOUN, M., ELLEOUZ, R., BEHI, J. & ATTIA, B. (1978). Histological types of nasopharyngeal carcinoma in an intermediate risk area. In *Nasopharyngeal Carcinoma: Etiology and Control*, de-The & Ito (eds). IARC Sci. Pub. no. 20, p. 13, IARC, Lyon.

CLIFFORD, P. & BEECHER, J.L. (1964). Nasopharyngeal cancer in Kenya: Clinical and environmental aspects. *Br. J. Cancer*, **18**, 25.

de-THE, HO, J.H.C. & MUIR, C.S. (1978). Nasopharyngeal carcinoma. In *Viral Infections of Humans: Epidemiology and Control*, Evans (ed) p. 539. Wiley, Chichester.

DOLL, R., MUIR, C.S. & WATERHOUSE, J.A. (1970). Cancer incidence in five continents, **2.** Springer-Verlag, Berlin.

EDWARDS, J.H. (1961). The recognition and estimation of cyclic trends. *Ann. Hum. Gen.*, **25**, 83.

HENLE, W. & HENLE, G. (1979). Seroepidemiology of the virus. In *The Epstein–Barr Virus*, Epstein & Achong (eds) p. 61. Springer-Verlag, Berlin.

HIRAYAMA, T. & ITO, Y. (1981). A new view of the etiology of nasopharyngeal carcinoma. *Prev. Med.*, **10**, 614.

HO, J.H.C. (1972). Nasopharyngeal carcinoma. *Adv. Cancer Res.*, **15**, 57.

KISSMEYER-NIELSEN, F., ANDERSEN, H., HAUGE, M., KJERBYE, K.E., MOGENSEN, B. & SVEJGAARD, A. (1971). HLA types in Danish Eskimos from Greendland. *Tissue Antigens*, **1**, 74.

KLEIN, G., (1979). The relationship of the virus to nasopharyngeal carcinoma. In *The Epstein–Barr Virus* Epstein & Achong (eds) p. 339. Springer-Verlag, Berlin.

KNOX, G. (1964). The detection of space-time interactions. *Appl. Statist.*, **13**, 25.

MINISTRY FOR GREENLAND. Yearly Report 1982. Copenhagen 1983.

NIELSEN, N.H., MIKKELSEN, F. & HANSEN, J.P.H. (1977). Nasopharyngeal cancer in Greenland: The incidence in an Artic Eskimo population. *Acta Path. Microbiol. Scand.* (*Sect A*), **85**, 850.

NIELSEN, N.H. & HART-HANSEN, J.P. (1982). Cancer incidence in Greendlanders. In *Circumpolar Health 81*, Harvald & Hart-Hansen (eds) (Proceedings of the 5th International Conference on Circumpolar Health, Oulu, Nordic Council for Arctic Medical Research) Report Series no. 33, p. 265.

OLD, L.J., BOYSE, E.A., OETTGEN, H.F., DE-HARVEN, E., GEERING, G., WILLIAMSON, B. & CLIFFORD, P. (1966). Precipitation antibodies in human serum to an antigen present in cultured Burkitt's lymphoma cells. *Proc. Natl Acad. Sci. USA*, **56**, 1699.

PIKE, M.C. & BULL, D. (1974). Knox test for space-time clustering in epidemiology. *Appl. Statist.*, **23**, 92.

SAEMUNDSEN, A.K., ALBECK, H., HANSEN, J.P.H. & 7 others (1982). Epstein–Barr virus in nasopharyngeal and salivary gland carcinomas of Greenland Eksimos. *Br. J. Cancer*, **46**, 721.

SCHMAUZ, R. & TEMPLETON, A.C. (1972). Nasopharyngeal carcinoma in Uganda. *Cancer*, **29**, 610.

SHANMUGARATNAM, K. (1982). Nasopharynx. In *Cancer Epidemiology and Prevention*, Schottenfeld & Fraumeni (eds) p. 536. Saunders, Philadelphia.

ZIPPIN, C., TEKAWA, I.S., BRAGG, K.U., WATSON, D.A. & LINDEN, G. (1962). Studies on heredity and environment in cancer of the nasopharynx. *J. Natl Cancer Inst.*, **29**, 483.


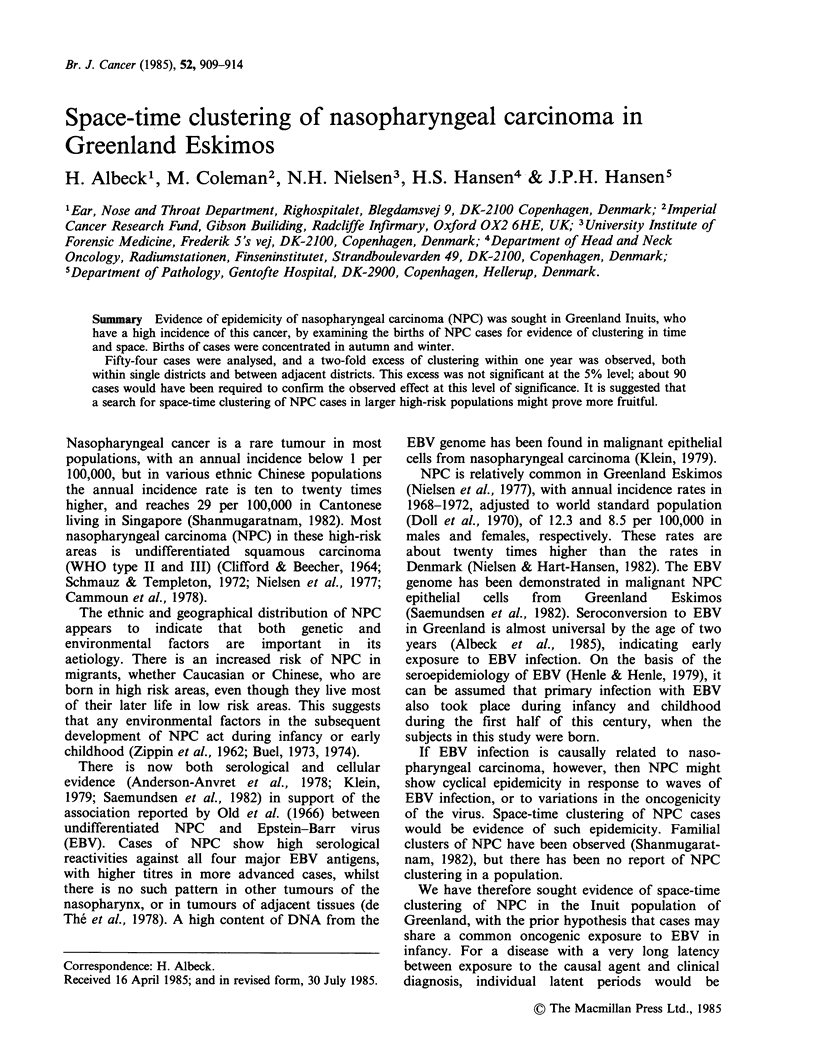

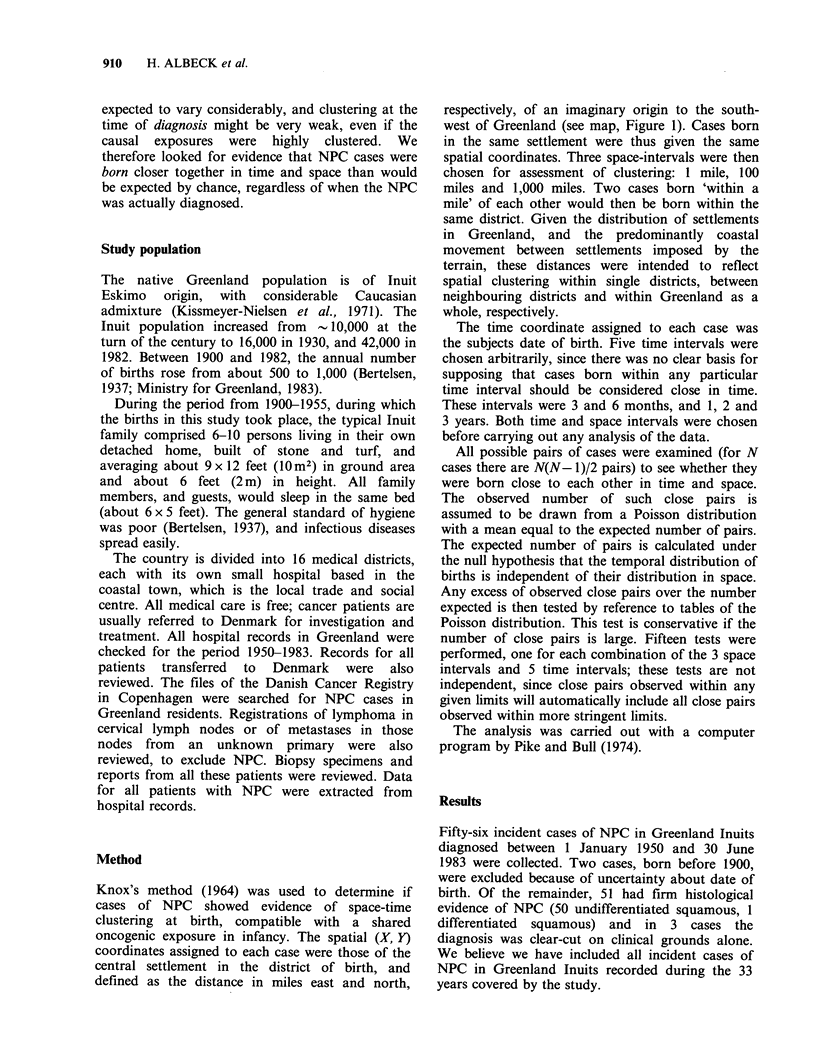

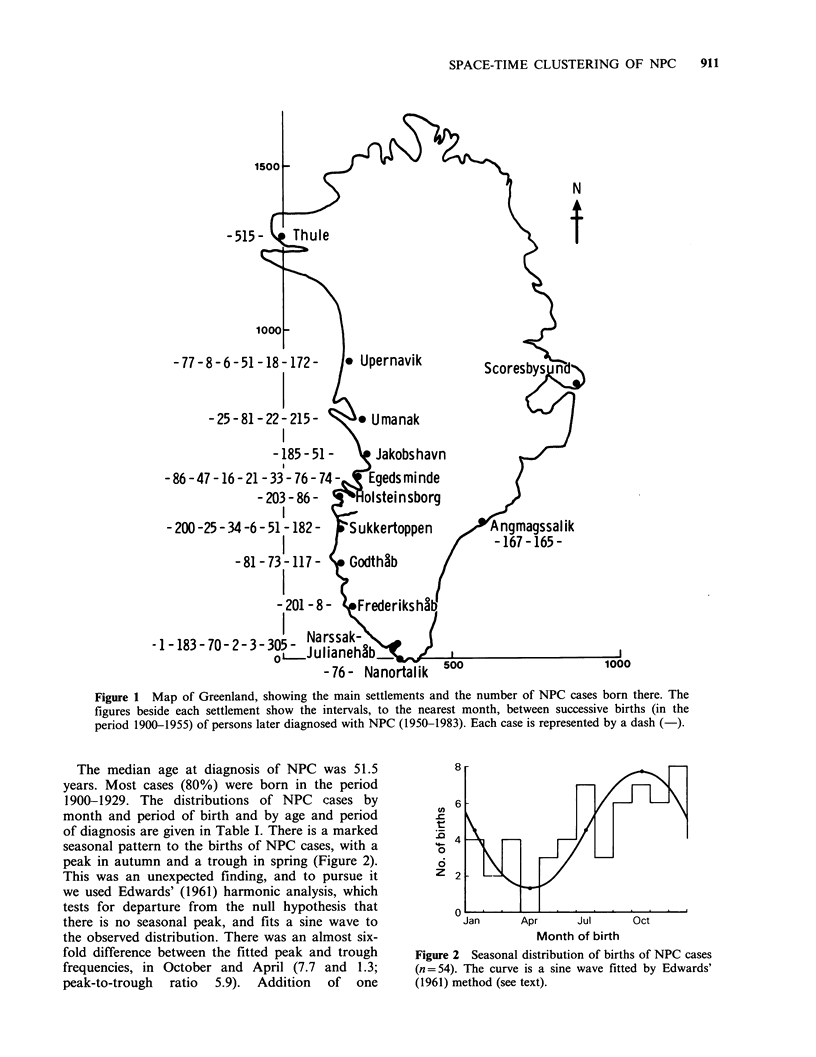

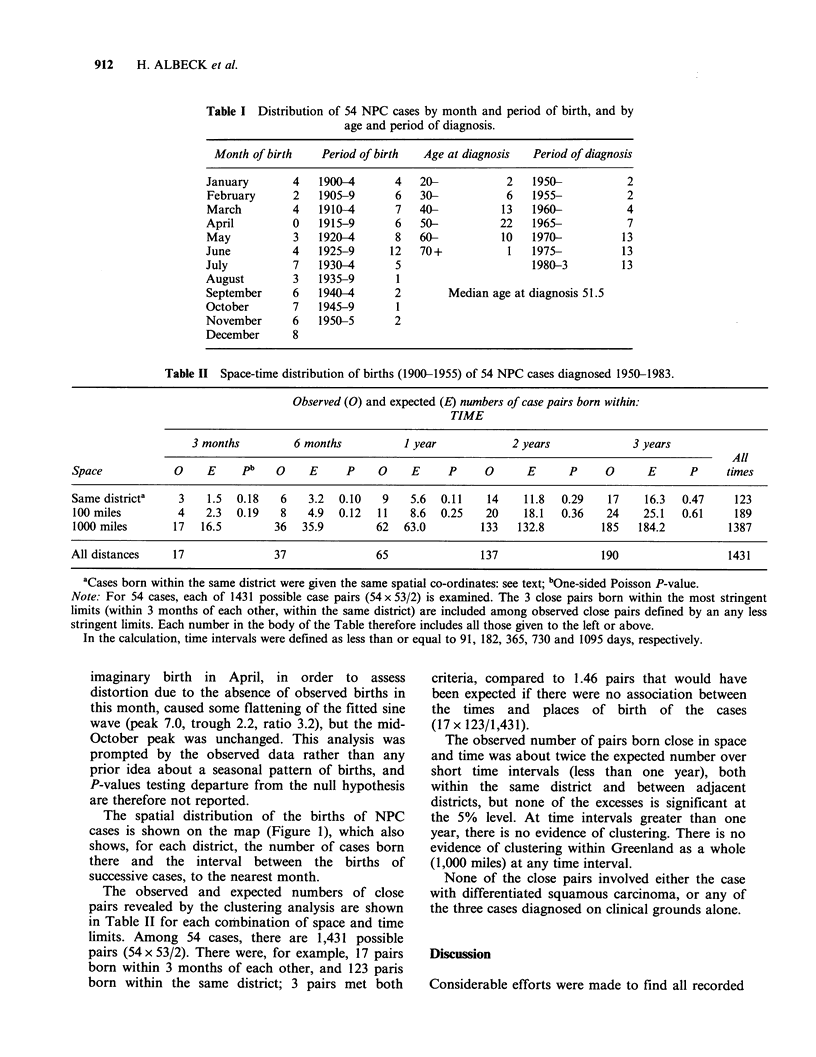

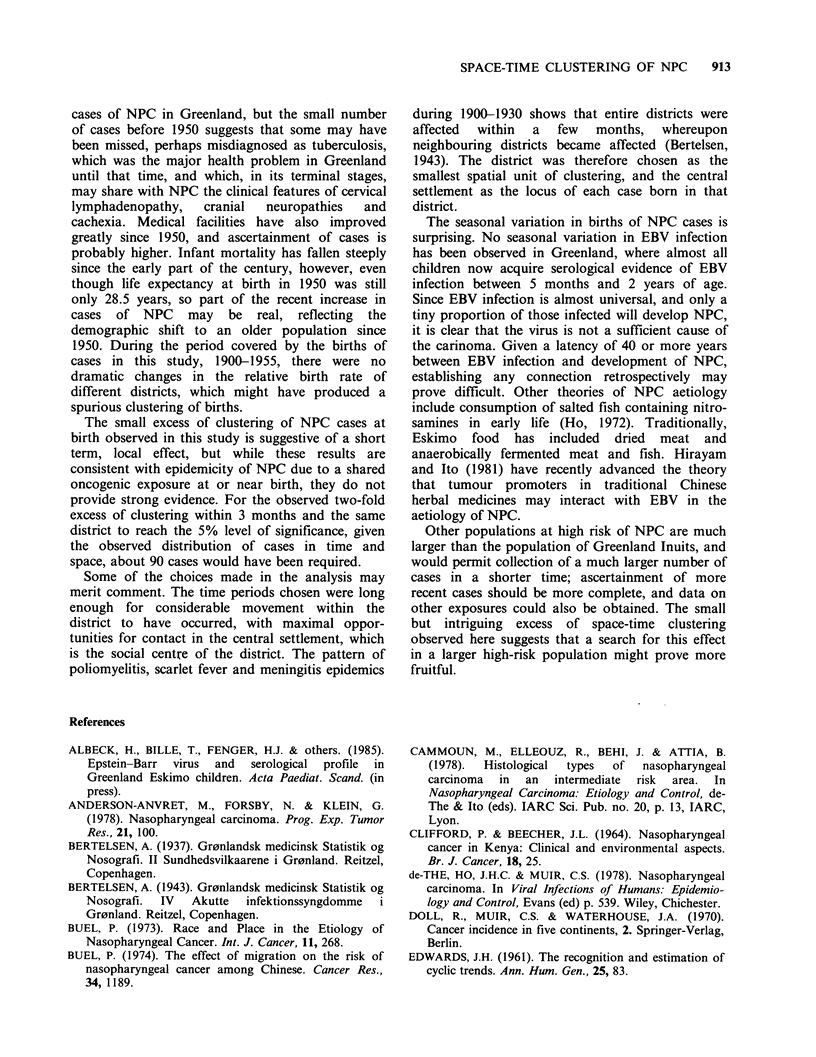

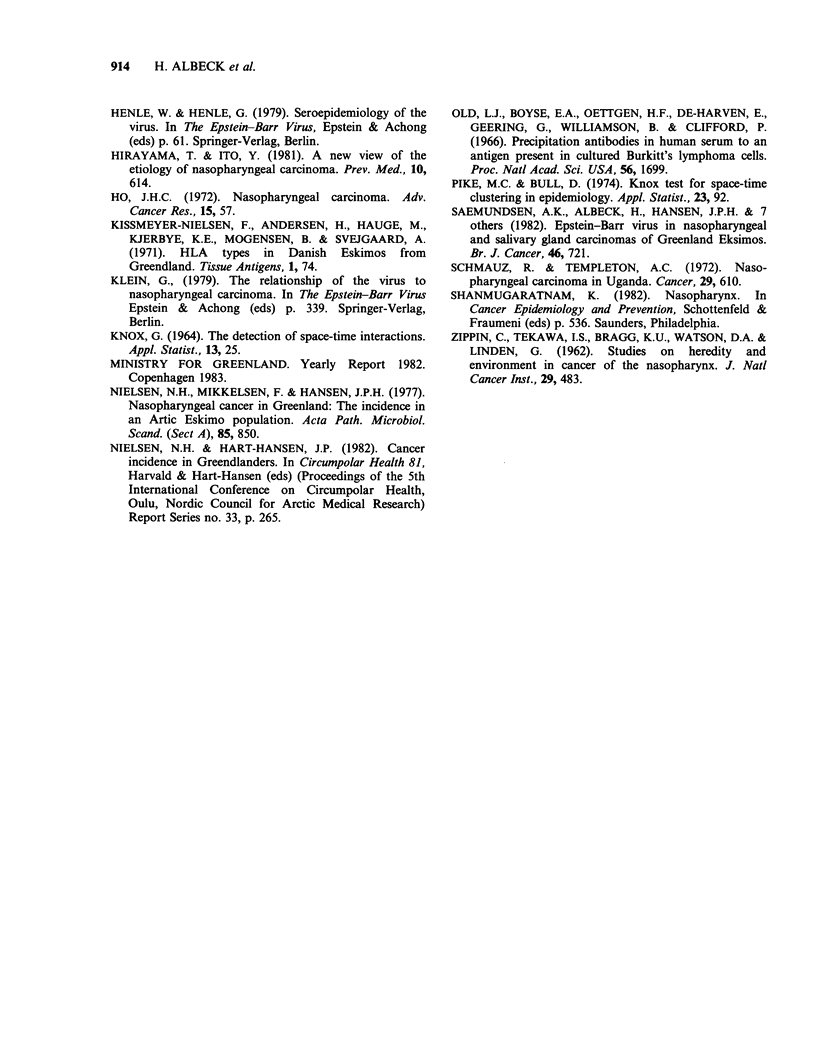


## References

[OCR_00464] Andersson-Anvret M., Forsby N., Klein G. (1978). Nasopharyngeal carcinoma.. Prog Exp Tumor Res.

[OCR_00479] Buell P. (1973). Race and place in the etiology of nasopharyngeal cancer: a study based on California death certificates.. Int J Cancer.

[OCR_00483] Buell P. (1974). The effect of migration on the risk of nasopharyngeal cancer among Chinese.. Cancer Res.

[OCR_00496] CLIFFORD P., BEECHER J. L. (1964). NASOPHARYNGEAL CANCER IN KENYA: CLINICAL AND ENVIRONMENTAL ASPECTS.. Br J Cancer.

[OCR_00511] EDWARDS J. H. (1961). The recognition and estimation of cyclic trends.. Ann Hum Genet.

[OCR_00522] Hirayama T., Ito Y. (1981). A new view of the etiology of nasopharyngeal carcinoma.. Prev Med.

[OCR_00527] Ho J. H. (1972). Nasopharyngeal carcinoma (NPC).. Adv Cancer Res.

[OCR_00531] Kissmeyer-Nielsen F., Andersen H., Hauge M., Kjerbye K. E., Mogensen B., Svejgaard A. (1971). HL-A types in Danish Eskimos from Greenland.. Tissue Antigens.

[OCR_00551] Nielsen N. H., Mikkelsen F., Hansen J. P. (1977). Nasopharyngeal cancer in Greenland. The incidence in an Arctic Eskimo population.. Acta Pathol Microbiol Scand A.

[OCR_00565] Old L. J., Boyse E. A., Oettgen H. F., Harven E. D., Geering G., Williamson B., Clifford P. (1966). Precipitating antibody in human serum to an antigen present in cultured burkitt's lymphoma cells.. Proc Natl Acad Sci U S A.

[OCR_00576] Saemundsen A. K., Albeck H., Hansen J. P., Nielsen N. H., Anvret M., Henle W., Henle G., Thomsen K. A., Kristensen H. K., Klein G. (1982). Epstein-Barr virus in nasopharyngeal and salivary gland carcinomas of Greenland Eskimoes.. Br J Cancer.

[OCR_00582] Schmauz R., Templeton A. C. (1972). Nasopharyngeal carcinoma in Uganda.. Cancer.

